# Reconfiguring clinical pharmacists’ roles under China’s DRG payment system: perspectives from clinical pharmacists and physicians in multidisciplinary teams

**DOI:** 10.3389/fpubh.2026.1930532

**Published:** 2026-10-20

**Authors:** Juan Fu, Xiang Gao, Qingran Sun, Xiaoyu Xi, Jing Wang

**Affiliations:** Department of The Research Center of National Drug Policy & Ecosystem, China Pharmaceutical University, Nanjing, Jiangsu, China

**Keywords:** clinical pharmacist, DRG payment, multidisciplinary team, pharmaceutical care, qualitative, role expectation misalignment

## Abstract

**Purpose:**

With the implementation of Diagnosis-Related Group (DRG) payment reform in China, clinical pharmacists (CPs) are expected to play expanded roles in the multidisciplinary team (MDT). This study explored the perceptions and experiences of CPs and physicians regarding CPs’ roles and contributions in MDT. The findings provide evidence to support MDT collaboration and the further development of pharmaceutical care.

**Methods:**

Semi-structured interviews were conducted from October 2023 to June 2024. Participants were recruited using purposive and snowball sampling from two tertiary Grade A hospitals in Nanjing, China. The study included 11 CPs and 10 physicians. The interview data were analyzed using Colaizzi’s seven-step method, and the themes were extracted.

**Results:**

Four themes were identified: Perceived Roles, Recognized Value and Contribution, Collaborative Experience and Existing Barriers, Future Expectations and Suggestions. CPs played multiple roles and made diverse contributions within MDT, but physicians mainly recognized their clinical support and DRG-related administrative functions, with less awareness of their roles in improving healthcare quality and managing healthcare costs. Greater involvement of CPs in clinical practice is still needed.

**Conclusion:**

The value of CPs in promoting rational medication use and improving healthcare quality was widely recognized. However, their contribution to cost control under the DRG remained limited by differences in role perceptions, unclear role definitions, and insufficient interprofessional collaboration. The future should focus on implementing the Pharmacists Law and advancing pharmacy education reform. These systemic measures could strengthen the role of CPs within MDT.

## 1 Introduction

Diagnosis-Related Group (DRG) has been widely adopted worldwide as a healthcare payment mechanism. It is commonly used to control healthcare costs and improve the efficiency of healthcare services. By the end of 2024, DRG had been implemented across all national pooling areas in China. This reform has prompted public hospitals to place greater emphasis on healthcare costs, resource-use efficiency, and quality of care. Since 2025, local authorities have further advanced the implementation of DRG 2.0. Following the implementation of DRG, the revenue and expenditure structure of public hospitals in China has changed. The share of revenue and expenditure related to medical services and personnel has increased, while that related to pharmaceuticals and medical consumables has declined. Hospitals are also placing greater emphasis on investment in human resources and the value of medical services. Overall, the reform is driving Chinese hospitals to shift from scale expansion toward development focused on quality and efficiency (1–3).

Meanwhile, the effects of the DRG payment reform have gradually extended to clinical healthcare professionals. Previous studies have shown that the DRG reform can influence physicians’ clinical decision-making and prescribing practices through hospital performance management and cost-control mechanisms. It may encourage physicians to pay greater attention to healthcare resource use, treatment costs, and appropriate medication use (4). Therefore, the appropriateness and cost-effectiveness of medication use, as well as its impact on resource use, have become important concerns for hospital management.

In this context, clinical pharmacists (CPs), as key contributors to medication therapy management, also need to balance appropriate medication use with the efficient use of healthcare resources. Meanwhile, as the DRG payment reform advances in China, the involvement and roles of hospital pharmacists in clinical practice are also evolving. Studies have found that CPs in hospitals implementing DRG are more actively involved in medication management and pharmaceutical care (5). The role of CPs is evolving in response to both their traditional responsibilities and the new demands arising from the DRG payment reform. This shift has also increased the need for knowledge of health economics and DRG related practices (6).

An effective multidisciplinary team (MDT) brings the expertise of different healthcare professionals. They are considered a key strategy for improving healthcare quality and cost control (7, 8). As medication experts, CPs have seen changes in their work priorities and competency requirements following the implementation of the DRG payment reform. Building on their traditional roles in clinical pharmacy services and medication management, they are increasingly involved in healthcare cost management, clinical pathway optimization, and optimization of pharmacotherapy regimens. These changes have also increased the need for knowledge of health economics and DRG-related practices (5). Previous studies have shown that fully integrating CPs into MDT can reduce adverse drug events (ADE), improve clinical outcomes, contribute to lower healthcare costs, and improve medication management through medication review and therapy monitoring (7–10). Research in China has also shown that CPs in MDT contribute to both cost control and quality improvement under DRG through specialized pharmaceutical care (6, 7, 11). In terms of cost control, CPs participating in obstetric MDT improved medication management; their involvement was associated with shorter hospital stays and lower hospitalization costs (11). In terms of healthcare quality, CP-led medication interventions reduced ADE in older adult patients with hip fractures and improved clinical outcomes (7). By supporting clinical pathway development and pharmacoeconomic evaluation, CPs help hospitals control costs under DRG while maintaining healthcare quality. However, CPs and physicians hold different perceptions of CPs’ roles and contributions in MDT collaboration, which may contribute to role ambiguity and role conflict (2, 11).

Most studies in China have explored the roles and challenges of CPs from the perspective of CPs only (6). However, MDT relies on continuous interaction among healthcare professionals, and a single perspective cannot fully capture the collaborative process or the barriers to effective teamwork (9, 11). To our knowledge, although international research has examined CPs’ involvement in cost management, clinical pathway optimization, and pharmacotherapy optimization in the context of payment reform, research on changes in the roles of CPs under the DRG system in China remains limited. In particular, few studies have examined role reconfiguration from the perspectives of both CPs and physicians or explored how these roles are manifested in MDT.

Therefore, this study adopts the perspectives of both CPs and physicians to explore how they perceive the roles and practical value of pharmacists in MDT collaboration under China’s DRG payment reform. It also examines how the reform has shaped the roles of CPs and influenced the focus of their responsibilities. The study focuses on how both groups perceive CPs’ roles in areas such as improving healthcare quality and controlling costs, as well as differences between these perceptions. It also examines how these perceptions are reflected in CPs’ daily practice and team collaboration. The findings help understand the evolving roles of CPs and provide empirical evidence for optimizing MDT collaboration mechanisms and maximizing the contributions of CPs in DRG reform.

## 2 Methods

### 2.1 Overview

A descriptive phenomenological approach was adopted with CPs and physicians. Nonverbal cues, including facial expressions, tone of voice, and body language, were recorded throughout the interviews, thereby helping provide a more comprehensive understanding of participants’ perceptions and experiences. All participants have signed informed consent forms.

The research team consisted of 5 members with expertise in hospital pharmacy and policy research; 4 had experience in qualitative interviewing. We consistently reflected on emerging findings with critical thinking, engaged in discussions, and reached consensus. This iterative process helped minimize potential researcher bias and enhance the rigor of the analysis.

### 2.2 Sampling

The study was approved by the Ethics Committee of China Pharmaceutical University and was conducted in two tertiary Grade A hospitals in Nanjing, China. Nanjing was among the first cities in China to implement the DRG payment reform, which was officially launched on January 1, 2022, and has since reached a relatively stable stage of implementation. The participating hospitals have well-established clinical pharmacy services and MDT practices, providing an appropriate setting for examining the roles of CPs and their collaboration with physicians under the DRG payment reform. Participants were recruited using purposive and snowball sampling. Purposive sampling was used to recruit eligible CPs and physicians with diverse professional roles, years of experience, professional titles, and departmental backgrounds. Snowball sampling was then used to recruit additional participants through referrals until data saturation was achieved.

The inclusion criteria of study subjects were: (1) Clinical pharmacists or physicians; (2) Hold a nationally recognized professional practice license; (3) Have experience participating in MDT; (4) Have at least 2 years of clinical practice experience; (5) Provide written informed consent.

Interviews and analysis were conducted concurrently. After each interview, the audio recordings were transcribed and subjected to preliminary analysis. Newly collected data were continuously compared with existing codes and preliminary themes. The data were analyzed by one researcher and independently reviewed by another with experience in qualitative research. The recruitment of study subjects continued until data saturation was reached (no new themes and information were identified during interviews). A total of 21 participants were included in the study, comprising 11 CPs and 10 physicians.

### 2.3 Data collection

Data were collected from October 2023 to June 2024 through one-to-one, face-to-face semi-structured interviews conducted by one researcher. This approach is well suited to phenomenological research and facilitates an in-depth exploration of personal experiences.

The initial interview guide was developed through a review of the relevant literature, phenomenological interviewing principles, and group discussions. It was pilot-tested with two CPs and two physicians from a tertiary Grade A hospital in Nanjing. The guide was subsequently refined based on findings from the pilot interviews and feedback from qualitative research experts. Two interview guides were developed, one for CPs and the other for physicians. The guide for CPs focused on their roles, challenges, and perceptions of the DRG payment reform, whereas the guide for physicians explored their perceptions of CPs and experiences of interprofessional collaboration within MDT. The full interview guides are provided in the Supplementary Material. Example questions included: “What obstacles and challenges have you encountered in multidisciplinary team-based care?” and “Could you describe your experience collaborating with clinical pharmacists?”

During the interviews, the researcher practiced active listening, avoided interrupting participants or asking leading questions, and maintained a neutral stance throughout the interviews. Participants were encouraged to express their views freely, while nonverbal cues were observed and documented. Interviews usually lasted 30–45 min and were conducted in a private setting. With participants’ written consent, all interviews were recorded and transcribed verbatim for subsequent analysis.

### 2.4 Data analysis

Within 24 h after each interview, the audio recordings were transcribed verbatim. Field notes documenting nonverbal cues, including tone of voice, facial expressions, and body language, were integrated into the transcripts. Data were managed and analyzed using NVivo14 software. An inductive analytical orientation was adopted. Colaizzi seven-step method was used for phenomenological data analysis. An inductive approach was used to allow themes to emerge from participants’ experiences, perceptions, and perspectives, rather than from predefined analytical categories. Analysis involved seven steps: (1) Read all participants’ descriptions; (2) Extract significant statements relevant to the research questions; (3) Formulate meanings from the significant statements; (4) Code, categorize, and cluster meaning units with similar content to develop preliminary themes; (5) Provide a detailed description of the phenomenon based on the participants’ original statements; (6) Repeatedly compare, integrate, and refine the preliminary themes and their underlying meanings to develop the final themes and subthemes; (7) Return to participants for validation (12). Two researchers independently conducted the data analysis. The analysis process was also guided by experts. Any discrepancies were discussed among the research team until consensus was reached, and the final themes and subthemes were identified across the team. The Consolidated Criteria for Reporting Qualitative Research (COREQ) guidelines were followed (13) (Supplementary Material).

## 3 Results

### 3.1 Participant characteristics

21 participants took part in this study, including 16 females and 5 males. Of the 11 CPs and 10 physicians, 10 (90.9%) and 7 (70.0%), respectively, held intermediate titles or above; 8 held master’s degrees and 13 held doctoral degrees. The mean ages of CPs and physicians were 36.8 ± 5.8 and 35.1 ± 6.2 years, respectively; all participants had 2–27 years of work experience and represented 11 clinical departments. The demographic characteristics of the participants are presented in Table 1.

**Table 1:** Characteristics of the participants (*n* = 11).

No.	Gender	Age	Years of work	Educational background	Professional title	Department
CP1	Female	50	27	Doctoral degree	Chief pharmacist	Pharmacy
CP2	Female	35	9	Master’s degree	Attending pharmacist	Hematology
CP3	Female	36	14	Master’s degree	Associate chief pharmacist	Rheumatology and Immunology
CP4	Male	42	17	Doctoral degree	Associate chief pharmacist	Endocrinology
CP5	Female	35	12	Doctoral degree	Associate chief pharmacist	Pain Medicine
CP6	Male	39	13	Master’s degree	Attending pharmacist	Cardiology
CP7	Female	38	10	Doctoral degree	Associate chief pharmacist	Endocrinology
CP8	Female	38	8	Doctoral degree	Associate chief pharmacist	Neurology
CP9	Female	29	4	Master’s degree	Pharmacist	Gastroenterology
CP10	Male	31	3	Doctoral degree	Attending pharmacist	Psychosomatic Medicine
CP11	Female	32	3	Doctoral degree	Attending pharmacist	Oncology
PY1	Female	28	2	Master’s degree	Resident physician	Rheumatology and Immunology
PY2	Female	27	2	Master’s degree	Resident physician	Endocrinology
PY3	Female	36	8	Doctoral degree	Associate chief physician	Rheumatology and Immunology
PY4	Male	31	3	Master’s degree	Resident physician	Endocrinology
PY5	Female	41	14	Doctoral degree	Attending physician	Cardiology
PY6	Male	46	22	Doctoral degree	Associate chief physician	Endocrinology
PY7	Female	34	9	Master’s degree	Attending physician	Nephrology
PY8	Female	42	17	Doctoral degree	Associate chief physician	Neurology
PY9	Female	34	10	Doctoral degree	Attending physician	Gastroenterology
PY10	Female	32	3	Doctoral degree	Attending physician	Psychosomatic Medicine

CP: Clinical Pharmacist; PY: Physician.

### 3.2 Themes on the role positioning and practical value of clinical pharmacists

The findings showed that both CPs and physicians shared common understandings while also expressing divergent perceptions regarding the roles, practical value, and future development of CPs within MDT. Four themes were identified from the data (Table 2).

**Table 2:** Themes and subthemes of clinical pharmacists’ roles and value in MDT under the DRG system.

Theme	Subtheme	Clinical pharmacists’ perspective	Physicians’ perspective
Perceived roles	Clinical treatment collaborator	√	√
	Rational medication guardian	√	√
	Cost management contributor	√	/
	Pharmaceutical knowledge disseminator	√	√
	Quality improvement facilitator	√	√
Recognized value and contribution	Improve the quality of healthcare	√	√
	Control healthcare costs	√	√
	Cost control has limited value	√	√
Collaborative experience and existing barriers	Deficiencies in relevant systems	√	/
	Lack of individual competence	√	/
	Social cognitive biases	√	/
	Inadequate objective conditions	√	/
	Work arrangements	/	√
	Level of engagement	/	√
	Professional competence	/	√
	Communication	/	√
	Medication recommendations	/	√
Future expectations and suggestions	Improve national policies and systems	√	/
	Increase hospitals’ commitment to this issue	√	/
	Promote the multidimensional development of individuals	√	/
	Strengthen educational guidance in higher education institutions	√	/
	Deepen collaboration	/	√
	Enhance personal skills	/	√
	Strengthen patient management	/	√
	Improve communication and interaction	/	√

#### 3.2.1 Perceived roles

CPs perceived themselves as indispensable clinical partners who collaborated with physicians through ward rounds, multidisciplinary discussions, and the development of individualized medication plans, providing professional recommendations to optimize medication therapy. As one pharmacist stated, their work often takes the initiative to advise physicians on medication use: “Most medication recommendations are initiated by pharmacists themselves.” (CP10).

The DRG payment reform has not altered the fundamental role of CPs as clinical treatment collaborators. However, some participants noted that clinical medication decisions under the DRG system should place greater emphasis on the cost-effectiveness of medications while ensuring treatment effectiveness.

“For pharmacists, DRG is still a useful leverage point… It may provide a good entry point for implementing and putting our recommendations into practice.” (CP5)

“Because the health insurance system has strict price controls, pharmacists are also quite familiar with medication prices and can help us select more appropriate medications.” (PY1)

Meanwhile, participants generally highly recognized CPs’ role in promoting rational medication use and disseminating pharmaceutical knowledge. Physicians fully recognized CPs’ key role in medication safety, patient education, and pharmaceutical knowledge dissemination.

“Clinical pharmacists review our medication orders. They check whether the prescriptions are appropriate, identify duplicate medications, and look for contraindications. They carefully review all of these issues.” (PY5)

“He (clinical pharmacist) communicates with patients. If they have any questions about their medications, he can provide counseling and education.” (PY9)

Drawing on their clinical practice, clinical pharmacists emphasized their important role in ensuring medication safety through reviewing medication orders and monitoring therapeutic outcomes and adverse drug reactions. One CP described identifying and correcting the following medication order error: “Just yesterday, I found a medication order for olanzapine. The intended dose was 1.25 mg, but the physician prescribed 1.25 tablets instead. That resulted in a fivefold dosing error (spoken emphatically).” (CP3)

Following the implementation of DRG, some participants noted that the focus of appropriate medication use has expanded to include health insurance coverage and payment conditions, medication costs, and the cost-effectiveness of medication use. As one CP explained, “Before DRG, there was no DRG… We only needed to follow the guidelines. If a treatment was effective, we would recommend it… But now, we also have to consider the cost-effectiveness of the medication.” (CP7) Another CP similarly noted that after the implementation of DRG, “we pay somewhat more attention to the price than before.” (CP11)

In addition, some CPs were also involved in training and providing guidance on DRG and health insurance policies. These activities covered appropriate medication use, health insurance payment conditions, and prescription review. One CP stated, “After DRG was introduced, pharmacists needed to provide relevant training for physicians… It was essentially about helping physicians better navigate DRG-related requirements.” (CP9)

However, both CPs and physicians differed markedly in their views of CPs’ emerging role as Cost management contributors under the DRG payment reform. Some CPs have begun to incorporate cost control into their pharmaceutical practice. They are involved in medication substitution, medication de-escalation, and the analysis of DRG cases with excessive expenditures.

“When DRG was first implemented, on the one hand, we mainly focused on how to help the clinical departments achieve a positive financial margin… Within this group, we tried to identify opportunities to recommend medication substitutions or simplification, so as to achieve greater savings.” (CP5)

Meanwhile, some CPs have begun to participate in developing medication treatment pathways for priority disease categories under the DRG system. They help control medication-related costs by promoting standardized medication regimens.

“Our department established a medication therapy management team… We identified the priority disease categories under the DRG payment system and developed medication treatment pathways for these conditions… These pathways were intended to complement and improve the medication regimens in the existing clinical pathways.” (CP4)

These practices suggest that DRG has prompted some CPs to move beyond traditional individual medication review and pay greater attention to medication selection, cost control, and resource allocation within clinical pathways.

However, this shift in role was not universally recognized among all CPs. Some participants reported that DRG had limited impact on their day-to-day work.

“DRG does not seem to have had much impact on my work… I do not pay much attention to DRG in my daily work.” (CP6)

In contrast, physicians had a more limited perception of CPs’ role in cost control. One physician stated, “At present, we do not really have such involvement. We mainly monitor whether our DRG cases fall into lower- or higher-weighted groups ourselves. Physicians are responsible for paying attention to this.” (PY10). Another physician noted, “The main decisions under DRG should still be made by physicians. Pharmacists can mainly help us review the medications we use, particularly their indications and contraindications.” (PY4)

Therefore, although some CPs have begun to take on DRG-related cost management responsibilities, this role has not yet been clearly and consistently recognized among physicians.

This misalignment in role perceptions was also evident in relation to quality improvement. CPs reported actively evaluating, analyzing, and optimizing medication use. They also contributed to clinical comprehensive medication evaluations, providing evidence to support clinical decision-making and improve healthcare quality. However, physicians had limited awareness of these contributions. They primarily viewed CPs as providers of clinical support, and only one physician briefly mentioned that: “At the beginning of each month, clinical pharmacists tell us which centrally procured medications our department should prescribe. They also remind us of important considerations for using these medications.” (PY1)

#### 3.2.2 Recognized value and contribution

The value of CPs in improving healthcare quality was widely recognized. CPs contributed to individualized treatment planning, therapeutic drug monitoring, and adverse drug reaction management. These activities were perceived to improve the appropriateness of medication use and enhance patient safety. “For transplant patients, we use therapeutic drug monitoring and other approaches to provide individualized medication management. This helps optimize medication use in clinical practice.” (CP2)

Physicians also recognized CPs’ contributions. They reported that CPs helped reduce prescribing errors and prevent potential medication risks through medication order review. As one Physician stated: “We usually prescribe trihexyphenidyl together with injectable haloperidol to prevent extrapyramidal symptoms. On one occasion, several of us forgot to prescribe it after ordering haloperidol. The clinical pharmacist noticed the omission and reminded us. That helped ensure the patient’s safety.” (PY10)

In the context of the DRG payment reform, some participants perceived that CPs’ traditional role in promoting appropriate medication use has taken on more explicit expectations regarding cost and resource use. CPs need to consider not only medication safety and effectiveness but also healthcare costs and health insurance payment requirements when selecting or substituting medications and optimizing medication regimens.

“Before DRG, we only needed to follow the guidelines… But now, we have to consider the cost-effectiveness of medications. If a medication is included in centralized procurement, we prioritize its use. The same applies to medicines included in national price negotiations.” (CP7)

However, the two professional groups differed considerably in their perceptions of CPs’ role in cost control, a core objective of the DRG reform. Some CPs described actively contributing to healthcare cost control by optimizing medication regimens.

“For originator drugs, I usually remind Physicians to consider whether a switch is appropriate. For example, when some patients have passed the acute phase, and cost control is a consideration, could we switch to centrally procured medications?” (CP9)

This difference in perceptions was also reflected in the extent to which participants were involved in DRG-related activities. Some CPs had incorporated DRG into their routine pharmaceutical practice. They contributed to cost control by analyzing DRG cases with excessive expenditures, optimizing medication regimens, and developing clinical medication pathways. In contrast, some physicians still viewed DRG as primarily the responsibility of clinical departments, with CPs mainly involved in appropriate medication review and supportive tasks.

“We are involved in reviewing the medication use and clinical pathways across all our wards, and we also participate in their implementation.” (CP1)

“At present, DRG-related decisions are still mainly managed by the physicians themselves.” (PY6)

But Physicians had limited recognition of CPs’ contributions to cost control. Most Physicians perceived this role simply as recommending lower-cost medications. As one Physician explained: “For some medication choices, she (Physician) can provide suggestions on which option offers better cost-effectiveness and is more appropriate.” (PY9)

However, CPs perceived their contributions to healthcare cost control as extending beyond medication regimen optimization. They highlighted their roles in analyzing DRG cases with excessive expenditures, conducting comprehensive clinical medication evaluations, and developing clinical medication pathways.

“We applied for a departmental innovation project this year (proudly). It focused on DRG medication reconciliation and medication substitution, including switching from originator drugs to generic alternatives and replacing non-essential medicines with essential medicines. We evaluated the impact of these interventions on patient outcomes and DRG surpluses through standardized medication management pathways. Based on both hospital indicators and our own evaluation metrics, we saw substantial improvements.” (CP5)

In addition, some participants perceived that DRG had expanded the value of CPs beyond simple cost containment toward balancing cost and effectiveness. Through pharmacoeconomic evaluation, monitoring of therapeutic outcomes and adverse effects, and other activities, CPs provided a more comprehensive basis for clinical treatment decisions. One CP stated, “We need to analyze cases with DRG-related excess expenditures in our own department or other clinical departments… and provide further guidance on medication regimens.” (CP10)

This finding suggests that, under the DRG system, the professional value of CPs is becoming more closely linked to healthcare resource allocation and the evaluation of treatment value.

More importantly, all participants identified several practical barriers that limited CPs’ contribution to healthcare cost control. Since the implementation of the centralized drug procurement policy, medication expenditures have declined substantially and are no longer the primary contributor to DRG cost overruns. As a result, CPs’ economic value was perceived to be most apparent in cases involving budget overruns, whereas savings generated through rational medication use were considered to have only a modest impact on overall healthcare expenditures.

“Many medications are now included in centralized procurement programs, but new drugs continue to emerge. Physicians may still prefer to select these new drugs within the DRG payment framework. As long as the costs do not exceed the DRG limit, clinical pharmacists generally have limited ability to intervene in these prescribing decisions.” (CP7)

In addition, limited decision-making authority also constrained CPs’ influence. The current clinical decision-making system remains largely physician-led, with CPs traditionally positioned in a supportive role. Their relatively limited involvement in treatment decisions, together with the imbalance in professional authority, may hinder proactive participation and prevent CPs’ recommendations from being fully incorporated into clinical practice. Furthermore, the lack of timely and direct access to DRG expenditure data further weakened the effectiveness of cost-control efforts.

“I think clinical pharmacists are capable of carrying out this type of work. However, we currently do not have access to the relevant data.” (CP3)

#### 3.2.3 Collaborative experience and existing barriers

Overall, physicians described their collaboration experiences with CPs positively, while also acknowledging certain limitations. Physicians generally perceived CPs as well-integrated members of MDT and recognized their expertise, proactive engagement, clinical contributions, and effective communication.

“Having clinical pharmacists involved in clinical practice is helpful in many aspects, particularly in medication-related management.” (PY10)

However, such collaboration was often restricted to specific situations. Some physicians preferred to manage routine medication-related issues independently, suggesting that CPs had limited involvement in routine clinical practice. One physician explained: “For some relatively straightforward diseases, we usually select treatment plans based on our own experience. But for more complex, challenging, or potentially risky cases, we discuss the options together with clinical pharmacists.” (PY6)

Regarding professional competence, physicians perceived that CPs mainly provided recommendations focused on the rational medications use. Although CPs possess solid pharmaceutical knowledge, some newly employed may have limited clinical experience, which can sometimes result in a gap between their practice and clinical needs. Differences in specific medication-related decisions were also occasionally observed. As one physician described, CPs emphasized that antibiotic use should comply with guideline recommendations, whereas physicians focused more on the patient’s specific surgical condition.

“I thought that this patient had a relatively long surgery with deeper surgical involvement, so a stronger antibiotic might be needed for prophylactic infection prevention. However, from the clinical pharmacist’s professional perspective, antibiotic use should be more consistent with national and international guidelines. She (clinical pharmacist) reminded me that this approach might be overly aggressive and that we should follow guideline recommendations by starting with a less intensive antibiotic regimen first.” (PY9)

In contrast, CPs’ perspectives revealed deeper structural barriers. First, institutional deficiencies represented a fundamental barrier. CPs lacked clearly defined responsibilities within the DRG framework and sufficient decision-making authority, creating a situation in which they were expected to take responsibility without having corresponding authority. One CP expressed frustration, stating: “We actually only provide recommendations. The final choice and decision-making authority still belong to the patients or physicians.” (CP11)

This dilemma of having responsibilities without corresponding decision-making authority was particularly evident in DRG-related work. Although some CPs had begun to participate in medication substitution, the analysis of cases with excessive expenditures, and clinical pathway optimization, decision-making authority over DRG-related clinical care remained largely with physicians and clinical departments. As one physician noted, “The main decisions under DRG should still be made by physicians. Pharmacists can mainly help us review the medications we use, particularly their indications and contraindications.” (PY4)

Just as one CP described, this sense of frustration became more apparent when encountering resistance: “You want to make a difference, but you do not have the authority. I can tell physicians that certain medications are under key monitoring and should not be prescribed arbitrarily. However, if physicians still choose to use them, we have limited ability to intervene from the perspective of rational medication use.” (CP7)

This also means that, although DRG has prompted some CPs to take on greater responsibilities for cost control and resource optimization, whether their recommendations are incorporated into clinical decision-making still depends on their acceptance by physicians and clinical departments.

Meanwhile, the lack of reimbursement mechanisms and financial incentives for pharmaceutical services further discouraged CPs’ active participation in MDT. As one CP described: “When pharmaceutical services are not reimbursed, we are essentially providing unpaid services while still being expected to complete all the related work.” (CP7)

Second, misperceptions regarding CPs’ roles represented another important barrier. CPs faced misunderstanding from physicians. Some physicians showed limited recognition of CPs’ roles and even expressed resistance, perceiving CPs’ recommendations on rational medication use as “unnecessary interference.” One CP described this sense of distrust with frustration: “There is often a lack of understanding from physicians regarding cost control, centralized procurement medications, and rational medication use (helplessly). When we identify inappropriate prescribing or medication use, some physicians may think, ‘Why is the pharmacy department doing this again? Is it really necessary to be so strict?’ (excitedly)” (CP8)

These misperceptions were not only present within the healthcare team but also extended to patients. Patients had limited awareness of CPs and often relied primarily on physicians for medication-related issues, resulting in inadequate recognition of their role in providing individualized medication guidance.

“Perhaps because clinical pharmacists are still a relatively small and less familiar professional group, patients may not fully recognize us. They may not have a clear understanding of what we actually do in clinical practice.” (CP2)

In addition, practical constraints further limited the efficiency of collaboration. Insufficient human resources were identified as a common concern by both CPs and physicians. As one CP described, CPs were responsible for a wide range of tasks, including ward rounds, prescription review, medication education, and therapeutic drug monitoring. The cumulative workload often exceeded their capacity. One CP commented: “Clinical work is extremely demanding and involves many routine tasks. It is difficult to complete everything (disappointingly), and sometimes some work has to be carried over to the next day.” (CP3)

Insufficient support from information systems further increased CPs’ workload. For example, poor interoperability between CPs workstations and other hospital information systems prevented efficient data sharing and utilization.

“Our hospital has a clinical pharmacist workstation, but for information such as DRG grouping, we still have to access the physicians’ system. The information systems are not well integrated with clinical pharmacists’ workflow.” (CP5)

#### 3.2.4 Future expectations and suggestions

CPs mainly called for stronger institutional support and greater recognition of their professional value. They emphasized the need for more robust national policy support, including the enactment of a national Pharmacists Law to clearly define CPs’ legal responsibilities and professional status, as well as the establishment of an appropriate reimbursement mechanism for pharmaceutical services.

“Providing pharmaceutical services without reimbursement is simply not sustainable. For example, if the reimbursement for pharmaceutical services during a patient’s hospital stay is only CNY 10, that might be enough if only one intervention is needed over 7 or 8 days. But if pharmaceutical care is required every day, it simply isn’t sustainable.” (CP7)

At the organizational level, CPs expected greater decision-making authority and better information system support. They also hoped for intelligent information systems capable of providing real-time alerts when DRG expenditure approached predefined thresholds. For example, a CP advocated granting CPs greater authority to intervene in medication management and provided an example of how this could be implemented: “Unless clinical pharmacists are given greater authority, it is difficult for us to make a real impact. I once heard about a hospital that established a quality control center involving the medical affairs department, the medical supplies department, and the pharmacy department. There, clinical pharmacists’ professional recommendations could directly inform quality management actions, including disciplinary actions.” (CP7)

In addition, some CPs emphasized the importance of access to DRG-related data and digital tools. They hoped to obtain real-time information on departmental costs, case groupings, and medication use. This would allow them to apply their pharmaceutical expertise more effectively to the analysis of DRG cases with excessive expenditures, optimization of medication regimens, and cost control.

One CP noted that access to relevant data would allow CPs to identify areas of excessive expenditure and opportunities for medication adjustment. Another CP suggested using information systems or intelligent tools to provide real-time alerts on DRG-related expenditures and reduce the burden of manual analysis.

These findings suggest that, as CPs become more involved in DRG-related activities, information systems are no longer merely tools for improving work efficiency. They are also becoming an important foundation for expanding CPs’ professional roles.

Regarding professional development, CPs hoped to expand the scope of their clinical services through clinical pharmacist-led outpatient clinics, chronic disease management, and follow-up care. They also expected these activities to strengthen patient engagement and better demonstrate their professional contribution. In addition, they hoped to participate more actively in clinical research to generate evidence for pharmacy practice.

In contrast, physicians expressed broader and more specific expectations regarding collaboration with CPs. They expected CPs to act as a liaison between clinical teams and the Pharmacy and Therapeutics Committee, communicating clinical medication needs in a timely manner and facilitating improvements in medication supply and formulary approval processes.

“We rely on clinical pharmacists to communicate our medication needs to the pharmacy department. For example, some medications under the centralized procurement program are unavailable because their prices are so low that manufacturers are unwilling to continue production.” (PY6)

Physicians also expected CPs to contribute more to hospital management under the DRG payment reform, including providing early warnings of potential DRG budget overruns, assisting with reimbursement appeals, and supporting related administrative documentation.

“It would be more appropriate for clinical pharmacists to help departments with some administrative tasks. For example, if the DRG system flags medication-related compliance issues, CPs could play a role in the reimbursement appeal process. I think that would be very beneficial.” (PY9)

In addition, physicians expected CPs to provide targeted training on the latest evidence in pharmacotherapy. “would be helpful if clinical pharmacists could provide regular training on different diseases and related medications. For example, they could introduce new drugs and the latest international evidence in pharmacotherapy. We cannot always keep up with these developments in our daily clinical practice, so their support would be invaluable.” (PY4)

Regarding professional competence, physicians recommended that CPs strengthen their clinical expertise and pursue subspecialized practice, allowing them to better understand the clinical reasoning and needs of specific departments. One physician suggested: “Clinical knowledge is essential for clinical pharmacists. They should develop expertise in selected specialties rather than attempting to cover all clinical departments. With sufficient experience in these areas, clinical pharmacists can better understand physicians’ clinical reasoning and decision-making processes.” (PY6)

In addition, physicians hoped that CPs could play a greater role in patient management, extend multidisciplinary collaboration, and support medication management across the entire continuum of care. They also expected CPs to facilitate more timely and effective communication among physicians, CPs, and patients.

## 4 Discussion

To our knowledge, this is the first qualitative exploration to examine how CPs’ roles and value are perceived in MDT collaboration under the DRG payment reform from the perspectives of both CPs and physicians. Our findings indicate that, as the DRG payment reform continues to advance, the role of CPSs is not being completely redefined. Rather, building on their existing responsibilities in promoting appropriate medication use and supporting clinical treatment, CPs are increasingly focusing on pharmacoeconomics, healthcare costs, and resource use (14). Although both CPs and physicians recognized CPs’ contributions to pharmaceutical care quality and appropriate medication use, they differed in their perceptions of CPs’ broader role under the DRG system, particularly in relation to cost control and economic value. Similar challenges have been reported in many countries undergoing healthcare payment reforms worldwide (15–18). This also suggests that payment reform may contribute to the reconfiguration of existing roles among CPs. However, a shared understanding of these roles has not yet been established. These findings provide insights into the key barriers limiting CPs’ engagement in MDT. They also offer practical guidance for improving hospital pharmacy service management, enhancing multidisciplinary collaboration, and advancing pharmaceutical care quality in the context of DRG reform.

Our findings indicate that both CPs and physicians recognized CPs as integral members of the MDT and acknowledged their important contributions to clinical practice and medication therapy. This finding is consistent with the global trend toward greater integration of CPs into clinical practice (6, 11, 18, 19). In recent years, pharmacy practice has evolved from a medication-centered to a patient-centered model. Consequently, CPs have become increasingly integrated into frontline clinical care. Their responsibilities have expanded beyond medication supply and management to include participation in ward rounds, consultations, prescription review, medication reconciliation, and individualized medication management. These expanded roles have contributed to safer and more appropriate medication use, making CPs indispensable members of the MDT (8, 11, 19, 20). This study further indicates that DRG did not alter this traditional role. Instead, it requires CPs to give greater consideration to the cost-effectiveness of medications and health insurance payment requirements when performing these tasks.

However, our findings also identified a clear divergence in CPs’ and physicians’ perceptions of the role of CPs in DRG. Even among pharmacists, recognition of this role was limited. Some CPs had begun to incorporate cost control, medication substitution, analysis of DRG cases with excessive expenditures, and optimization of clinical medication pathways into their pharmaceutical practice. In contrast, other CPs perceived that DRG had limited impact on their day-to-day work. Physicians, meanwhile, tended to perceive CPs’ DRG-related roles in terms of specific activities, such as medication selection, appropriate medication use, and cost control. This may be because CPs’ contributions to cost control are often less visible and primarily retrospective. Their work typically focuses on reviewing DRG budget overrun cases rather than proactively preventing avoidable costs or intervening during patient care. Consequently, these contributions are difficult to measure and may not be fully recognized by physicians (6, 21). In addition, decision-making authority over DRG-related matters remains largely with physicians and clinical departments. This may also limit the further development of pharmacists’ roles in this area. These differing perceptions of CPs’ contribution to cost control reflect the role ambiguity and role conflict accompanying their transition under DRG reform. According to role theory, unclear professional boundaries and expectations may lead stakeholders to hold different perceptions of professional responsibilities (22). In China, current policies emphasize CPs’ contributions to appropriate medication use and healthcare quality, whereas their responsibilities in cost control remain insufficiently defined. This may explain the differing perceptions of CPs and physicians regarding CPs’ contribution to cost control (23). Clarifying CPs’ responsibilities within the DRG system could facilitate interprofessional collaboration and foster a shared understanding of their professional responsibilities (24).

Although CPs and physicians differed in their perceptions of CPs’ role as cost managers, both acknowledged that CPs contributed to cost control, although the impact was considered limited. This suggests that CPs’ contribution to cost control is perceived primarily as a technical task rather than as a core institutional role (25). Specifically, physicians are able to directly observe the economic benefits generated by CPs’ interventions, such as recommending lower-cost medications through the national centralized procurement program and optimizing medication therapy (6). These visible outcomes make it easier for physicians to recognize the value of CPs’ contributions. However, multiple factors limit the full realization of this value. First, since the implementation of the national centralized drug procurement policy, drug expenditures have become a smaller component of overall healthcare spending and are no longer the primary driver of budget overruns (6, 26). Second, clinical decision-making remains patient-centered, with physicians prioritizing treatment strategies they consider most beneficial for patients when clinical benefit conflicts with cost-containment goals. Consequently, physicians perceive less need for CP involvement in cost control, reducing the adoption of pharmacists’ recommendations in clinical practice (11). Therefore, future assessments of CPs’ value should not be limited to medication cost savings. A value-based healthcare framework is needed to better capture CPs’ broader contributions to healthcare quality, patient outcomes, and cost efficiency (27).

Although the role of CPs in MDT is widely recognized, the full realization of their professional value remains constrained by multiple factors. CPs placed greater emphasis on insufficient institutional support, including the lack of a Pharmacists Act, prescribing authority, and reimbursement mechanisms for pharmaceutical care, which limited professional recognition and resource availability (6, 11, 28, 29). In contrast, physicians emphasized practical barriers to collaboration, including inadequate pharmacist staffing, the limited applicability of CPs’ recommendations to complex clinical cases, and communication delays. Consistent with previous studies, healthcare professionals recognized CPs’ value in addressing medication-related issues but showed limited support for their broader strategic role (30, 31). It is worth noting that the expansion of CPs’ responsibilities under the DRG system has not been fully accompanied by corresponding decision-making authority and access to information. This may further limit their ability to realize their professional value. In fact, the barriers to collaboration perceived by physicians largely reflect the institutional constraints identified by CPs. Together, these findings suggest that integrating CPs more fully into MDT requires improvements at the institutional, organizational, and professional levels.

To address these challenges, recommendations from both CPs and physicians collectively suggested a multi-level improvement framework encompassing institutional, organizational, and educational domains. For policy, a Pharmacists Act and appropriate reimbursement mechanisms for pharmaceutical care are essential to strengthen recognition of CPs’ professional role and contributions. For organizational healthcare institutions should strengthen information systems and pharmacy workforce capacity while expanding CPs’ authority in medication management to support their involvement in rational medication use and DRG management. For educational, efforts should focus on strengthening clinical pharmacy training programs and developing interprofessional communication skills to enhance CPs’ clinical competence. CPs emphasized the need for greater professional autonomy and recognition of their clinical contributions, while physicians more often perceived CPs as facilitators who support the optimization of clinical workflows. Such differences in role expectations have also been observed in international research (16). Therefore, the deeper integration of CPs into MDT requires not only institutional support and adequate resources but also continuous collaboration, capability development, and improved digital infrastructure to foster shared role understanding and collaborative goals (32).

From a broader perspective, this study provides insights into how the roles of CPs are changing under the DRG payment reform and the specific healthcare system context in which these changes occur. In China, the DRG/DIP payment reform has been implemented alongside centralized procurement of pharmaceuticals, health insurance payment policies, and performance assessments of public hospitals. These policy mechanisms have further integrated considerations of healthcare costs, medication cost-effectiveness, and healthcare resource use into clinical pharmacy practice. At the same time, physician-led clinical decision-making remains common in China, creating a gap between the changing responsibilities of CPs and their actual decision-making authority. Our findings therefore not only confirm some common barriers previously reported in international pharmacy practice research but also illustrate how these challenges manifest within China’s specific payment reform and healthcare system context. For other healthcare systems undergoing payment reform, these findings suggest that payment reform should not focus solely on economic incentives. It should also consider CPs’ responsibilities, professional competencies, and mechanisms for their participation in decision-making within MDT. Such considerations may help better align the requirements of payment reform with professional pharmacy practice.

### 4.1 Limitations

However, this study has several limitations. First, the sample size may be considered relatively small from a quantitative research perspective. But the sampling strategy and data collection process were consistent with qualitative research methodology, and data saturation was achieved among participants. Second, participants were recruited from only two tertiary Grade A hospitals in Nanjing, which may limit the transferability of the findings to other healthcare settings and regions. Third, as this qualitative study relied on participants’ self-reported perspectives, the findings may have been subject to social desirability bias. Finally, this study explored MDT collaboration from the perspectives of CPs and physicians only. Other important stakeholders, such as nurses and hospital administrators, were not included. Therefore, the findings represent the experiences of two key professional groups and may not fully capture the perspectives of all MDT members.

## 5 Conclusion

We provide insights into the roles and value of CPs in MDT during China’s DRG payment reform by incorporating the perspectives of both CPs and physicians. The findings indicate that, under the DRG system, CPs continue to fulfill their traditional responsibilities, while some of their roles have gradually expanded to include pharmacoeconomics, cost management, and resource use. However, this role change has not been consistently recognized and remains constrained by factors such as decision-making authority and institutional support. Therefore, payment reform should be aligned with adjustments in CPs’ roles, professional competencies, and MDT collaboration mechanisms. This would enable CPs to participate more effectively in clinical decision-making and the optimization of healthcare resource use.

## Data Availability

The raw data supporting the conclusions of this article will be made available by the authors, without undue reservation.
