# Supplementary material for: Reconfiguring clinical pharmacists’ roles under China’s DRG payment system: perspectives from clinical pharmacists and physicians in multidisciplinary teams
Source: Front Public Health. 2026 Oct 20;14:1930532. doi: 10.3389/fpubh.2026.1930532 (PMC13727457; doi:10.3389/fpubh.2026.1930532)
Supplement: Supplementary file 1 [file Data_sheet_1.docx]

Supplementary Material

**S1--** **General Information Questionnaire and Interview Guide**

**S1-1 Clinical Pharmacist**

(1) Clinical Pharmacist General Information Sheet

Dear Professor,

Hello! Thank you for taking the time out of your busy schedule to participate in this interview!

This questionnaire is designed to collect general background information from participants. The information gathered will be used solely for this study, and we will keep all the details you provide strictly confidential, so please feel free to answer without any reservations. If you encounter any questions you do not understand while completing the questionnaire, please feel free to ask the research staff at any time. Finally, thank you once again for your generous support of this study!

**1. Gender:** ☐ Male	☐ Female

**2. Age:** years old

**3. Education:** ☐ Associate's degree	☐ Bachelor's degree	☐ Master's degree	☐ Doctorate and postdoctoral degree

**4. Years of experience:** years

**5. Department or division:**

**6. Professional Title:** ☐ Pharmacy Technician	☐ Pharmacist	☐ Senior Pharmacist	☐ Associate Chief Pharmacist	☐ Chief Pharmacist	☐ No Professional Title

(2) Clinical Pharmacist Interview Guide

1. Please share your views on the hospital’s policies and regulations regarding clinical pharmacists.

2. Please carefully reflect on your work experience. In terms of the overall clinical care process, in which stages of clinical care have you been involved in your actual work? What specific duties have you performed?

3. Please describe the medication usage and DRG profit and loss situation in the department where you are currently assigned.

4. Has the hospital’s implementation of the DRG payment system had an impact on your work?

5. Do you believe your work has any impact on the implementation of the DRG payment system?

6. From your perspective, how has the implementation of the DRG payment system affected the work of clinical physicians?

7. How do you think your work can help mitigate the impact of DRG on clinical physicians?

8. As a clinical pharmacist, what else do you think you can contribute in the future?

9. What obstacles and challenges have you encountered in multidisciplinary team-based care?

10. Do you have any suggestions for improving these obstacles and challenges that you can share?

**S1-2 Physician**

(1) Physician General Information Sheet

Hello! Thank you for taking the time out of your busy schedule to participate in this interview!

This questionnaire is designed to collect general background information from participants. The information gathered will be used solely for this study, and we will keep all the details you provide strictly confidential, so please feel free to answer without any reservations. If you encounter any questions you do not understand while completing the questionnaire, please feel free to ask the research staff at any time. Finally, thank you once again for your generous support of this study!

**1. Gender:** ☐ Male	☐ Female

**2. Age:** years old

**3. Education:** ☐ Associate's degree	☐ Bachelor's degree	☐ Master's degree	☐ Doctorate and postdoctoral degree

**4. Years of experience:** years

**5. Department or division:**

**6. Professional Title:** ☐ Medical Assistant	☐ Resident Physician	☐ Attending Physician	☐ Associate Chief Physician	☐ Chief Physician	☐ No Professional Title

(2) Physician Interview Guide

1. Please describe your understanding of the hospital’s policies and regulations regarding clinical pharmacists.

2. Please carefully reflect on your work experience. In terms of the overall clinical care process, in which stages do clinical pharmacists participate in practice? What specific duties do they perform?

3. Please describe the medication usage and DRG profitability in your current department.

4. Do you believe that the work of clinical pharmacists has an impact on the implementation of the DRG payment system?

5. How do you think the implementation of the DRG payment system has affected your work?

6. Do you believe that the work of clinical pharmacists helps mitigate the impact of DRG on you?

7. Could you describe your experience collaborating with clinical pharmacists?

8. What additional competencies do you think clinical pharmacists should possess? What other responsibilities should they undertake?

9. Do you have any suggestions for improving the work of clinical pharmacists that you would like to share?

**S2--COREQ for reporting qualitative studies**

**The checklist of the research**

Consolidated criteria for reporting qualitative studies (COREQ): 32-item checklist

| No Item | Guide questions/description | Answers |
| --- | --- | --- |
| **Domain 1: Research team and reflexivity** | | |
| Personal Characteristics | | |
| 1. Interviewer/facilitator | Which author/s conducted the interview or focus group? | Xiang Gao |
| 2. Credentials | What were the researcher’s credentials? E.g. PhD, MD | MD (Xiang Gao) or PhD (Qingran Sun) |
| 3. Occupation | What was their occupation at the time of the study? | Master's student (Xiang Gao) and Doctoral student (Qingran Sun) |
| 4. Gender | Was the researcher male or female? | Male (Xiang Gao) and Female (Qingran Sun) |
| 5. Experience and training | What experience or training did the researcher have? | Theoretical training in qualitative research methods and experience of the research team in clinical pharmacy, physician–pharmacist collaboration, MDTs, and DRG reform. |
| Relationship with participants | | |
| 6. Relationship established | Was a relationship established prior to study commencement? | No |
| 7. Participant knowledge of the interviewer | What did the participants know about the researcher? e.g. personal goals, reasons for doing the research | Reasons for doing the research |
| 8. Interviewer characteristics | What characteristics were reported about the interviewer/facilitator? e.g. Bias, assumptions, reasons and interests in the research topic | Reasons and interests in the research topic, awareness of bias |
| **Domain 2: study design** | | |
| Theoretical framework | | |
| 9. Methodological orientation and Theory | What methodological orientation was stated to underpin the study? e.g. grounded theory, discourse analysis, ethnography, phenomenology, content analysis | Phenomenology. |
| Participant selection | | |
| 10. Sampling | How were participants selected? e.g. purposive, convenience, consecutive, snowball | Purposive and Snowball. |
| 11. Method of approach | How were participants approached? e.g. face-to-face, telephone, mail, email | Face-to-face interview |
| 12. Sample size | How many participants were in the study? | 21 |
| 13. Non-participation | How many people refused to participate or dropped out? Reasons? | No participants refused to participate or withdrew from the study. |
| Setting | | |
| 14. Setting of data collection | Where was the data collected? e.g. home, clinic, workplace | Workplace. |
| 15. Presence of non-participants | Was anyone else present besides the participants and researchers? | No |
| 16. Description of sample | What are the important characteristics of the sample? e.g. demographic data, date | Yes, demographic data. |
| Data collection | | |
| 17. Interview guide | Were questions, prompts, guides provided by the authors? Was it pilot tested? | Yes |
| 18. Repeat interviews | Were repeat interviews carried out? If yes, how many? | No |
| 19. Audio/visual | Did the research use audio or visual recording to collect the data? | Audio recording |
| 20. Field notes | Were field notes made during and/or after the interview or focus group? | Yes |
| 21. Duration | What was the duration of the interviews or focus group? | 30-45 min |
| 22. Data saturation | Was data saturation discussed? | Yes |
| 23. Transcripts returned | Were transcripts returned to participants for comment and/or correction? | Yes |
| **Domain 3: analysis and findings** | | |
| Data analysis | | |
| 24. Number of data coders | How many data coders coded the data? | Two |
| 25. Description of the coding tree | Did authors provide a description of the coding tree? | No |
| 26. Derivation of themes | Were themes identified in advance or derived from the data? | derived from the data |
| 27. Software | What software, if applicable, was used to manage the data? | NVIVO 14 |
| 28. Participant checking | Did participants provide feedback on the findings? | Yes |
| Reporting | | |
| 29. Quotations presented | Were participant quotations presented to illustrate the themes / findings? Was each quotation identified? e.g. participant number | Yes |
| 30. Data and findings consistent | Was there consistency between the data presented and the findings? | Yes |
| 31. Clarity of major themes | Were major themes clearly presented in the findings? | Yes |
| 32. Clarity of minor themes | Is there a description of diverse cases or discussion of minor themes? | Yes |
